# A high-density SNP-based genetic map and several economic traits-related loci in *Pelteobagrus vachelli*

**DOI:** 10.1186/s12864-020-07115-7

**Published:** 2020-10-07

**Authors:** Guosong Zhang, Jie Li, Jiajia Zhang, Xia Liang, Tao Wang, Shaowu Yin

**Affiliations:** 1 College of Marine Science and Engineering, College of Life Sciences, Nanjing Normal University, Nanjing, 210023 China; 2 Key laboratory for physiology biochemistry and application, Heze University, Heze, 274015 Shandong China; 3 Co-Innovation Center for Marine Bio-Industry Technology, Lian Yungang, 222005 China

**Keywords:** ddRAD-seq, QTL mapping, Growth, Sex determination, Hypoxia tolerance

## Abstract

**Background:**

A high-density genetic linkage map is essential for QTL fine mapping, comparative genome analysis, identification of candidate genes and marker-assisted selection in aquaculture species. *Pelteobagrus vachelli* is a very popular commercial species in Asia. However, some specific characters hindered achievement of the traditional selective breeding based on phenotypes, such as lack of large-scale genomic resource and short of markers tightly associated with growth, sex determination and hypoxia tolerance related traits.

**Results:**

By making use of 5059 ddRAD markers in *P. vachelli,* a high-resolution genetic linkage map was successfully constructed. The map’ length was 4047.01 cM by using an interval of 0.11 cm, which is an average marker standard. Comparative genome mapping revealed that a high proportion (83.2%) of markers with a one-to-one correspondence were observed between *P. vachelli* and *P. fulvidraco*. Based on the genetic map, 8 significant genome-wide QTLs for 4 weight, 1 body proportion, 2 sex determination, and 1 hypoxia tolerance related traits were detected on 4 LGs. Some SNPs from these significant genome-wide QTLs were observably associated with these phenotypic traits in other individuals by Kompetitive Allele Specific PCR. In addition, two candidate genes for weight, Sipa1 and HSD11B2, were differentially expressed between fast-, medium- and slow-growing *P. vachelli*. Sema7a, associated with hypoxia tolerance, was induced after hypoxia exposure and reoxygenation.

**Conclusions:**

We mapped a set of suggestive and significant QTLs as well as candidate genes for 12 growth, 1 sex determination and 1 hypoxia tolerance related traits based on a high-density genetic linkage map by making use of SNP markers for *P. fulvidraco*. Our results have offered a valuable method about the much more efficient production of all-male, fast growth and hypoxia tolerance P. vachelli for the aquaculture industry.

## Background

High-resolution linkage maps have become indispensable to many genetic studies, such as fine-scale quantitative trait locus (QTL) mapping, mapping molecular marker-assisted selection (MAS), genome scaffolding and assembly (GSA) and comparative genomic analysis (CGA). For the construction of high-density genetic linkage maps, single-nucleotide polymorphism (SNP) markers representing the most abundant sources of variation in the genome are increasingly utilized [1]. Newly developed genotyping methods, such as reduced-representation sequencing, SNP arrays, and re-sequencing, have allowed for the discovery and simultaneous scoring of thousands of SNP markers from a single sequencing run for dozens of individuals. These techniques have already been applied in some fish species, such as *Ictalurus punctatus* [2], *Siniperca chuatsi* [3], *Scophthalmus maximus* [4], *Paralichthys olivaceus* [5], and *Megalobrama amblycephala* [6]. In addition, significant QTLs for weight, gender and disease resistance in these fish species have been detected. MAS using QTL analysis is the most effective breeding method for many animals and plant species and it is a direct choice for genotyping the control trait loci.

In Pelteobagrus genus, adult individuals of Pelteobagrus vachelli are the largest [7]. In Asia, the reason why it is a very popular commercial species is that it has relatively high yield together as well as an affordable price for consumers to buy [8]. This species is also known as the male parent of the new aquatic species ‘Huangyou-1 catfish’ (*P. vachelli* ♂ × *P. fulvidraco* ♀), which has advantages such as faster growth, a high feeding rate, and greater immunity when compared to its parent species [9]. As with a lot of other cultured fish species, the production of *P. vachelli* seedlings by catching wild parents and carrying out multiple generations of inbreeding has given rise to varying qualities and the degradation of economic characteristics, e.g., body length, weight, head length and condition factors. These problems result in farming production efficiencies and economic benefits that are difficult to guarantee, in addition to the declining competition for these goods in fish markets, which have seriously affected the sustainable development of the *P. vachelli* industry. Identification of molecular markers from important QTLs for growth-related characters could contribute to efficient breeding varieties, similar to several other aquaculture species, including *Lates calcarifer* [10], *Hypophthalmichthys nobilis* [11], *Oncorhynchus nerka* [12], and *O. tshawytscha* [13].

Fish species often exhibit significant sexual dimorphism [14]. Commercial fish species, such as *Dicentrarchus labrax* [15], *Hippoglossus hippoglossus* [16], and *Oreochromis niloticus* [17], display significant variations in size and growth rates between female and male individuals, substantially affecting their commercial value. Bagridae fish, the Pelteobagrus genus in particular, also exhibit significant sexual dimorphism [18, 19]. Females tend to grow more slowly than males and lower body weights (up to 30% less) have been reported at harvest time compared to the body weights of identical cohorts of males [20]. It’s worth noting that *Pelteobagrus fulvidraco* has achieved all-male fish lines [21], which is contributed by sex-associated markers for successfully breeding YY-male and YY-female [22]. Therefore, screening for sex-associated markers will shorten the time required for the development of all-male fish lines for aquaculture and will be helpful in elucidating the mechanisms of sex chromosome differentiation and sex determination [23].

Exposure to hypoxia induces both acute and chronic stress responses, which play important roles in the health of cultured organisms, such as growth, reproduction, immunity, and other energy demanding activities [24]. Many environmental factors (e.g., elevated temperatures, decomposition, algal blooms, and organic matter accumulation via faeces) will lead to rising biological oxygen demand and make the hypoxia status more serious for animals in the system. Living in hypoxic conditions can place selection pressure on genotypes which could tolerate hypoxia at the genomic level more [25], thus developing lines by MAS that are more tolerant to hypoxia is an economical as well as sustainable solution to the problem about aquaculture. However, progress in improving hypoxia tolerance in fish, including Bagridae fish, has been very slow due to the shortage of genetic and physiological knowledge of hypoxia tolerance traits. With fish, especially aquaculture species, genetic analysis of the QTLs associated with hypoxia tolerance is important for efficient MAS in fish, as has been demonstrated in *I. punctatus* [26, 27] and *O. niloticus* [28].

Although considerable work has been carried out in *P. vachelli* to increase production, some specific characteristics have continued to hinder the development of traditional phenotype-based selective breeding techniques, such as lacking in large-scale genomic resources as well as few markers that are tightly associated with growth-, sex determination- and hypoxia tolerance-related traits. MAS technology, which uses a series of selected markers that are connected to economical traits tightly, can be used to develop stress tolerant and fast growing lines more effectively [29]. As a result, it is of great necessity to start a selective breeding programme for *P. vachelli* to promote these economical traits. Comparing to existing RAD-Seq approaches, as a reduced-representation sequencing method, double digest restriction-site associated DNA sequencing (ddRAD-seq) is a way for de novo SNP discovery and genotyping in non-model species that is efficient and cost-saving [30]. This technique has already been applied in some fish, such as *Oncorhynchus kisutch* [31] and *Polyprion oxygeneios* [32]. For the specific characteristics of *P. vachelli*, the ddRAD-seq technique is a very effective and appropriate method for the MAS of a selective breeding programme.

In this study, a high-resolution genetic linkage map of *P. vachelli* was constructed using the ddRAD-seq technique for comparative genome mapping with assembled genomes of *I. punctatus* and *P. fulvidraco*, as well as for the fine mapping of economical QTL traits, including 13 growth-, 1 sex determination-, and 1 hypoxia tolerance-related trait. Kompetitive Allele Specific PCR (KASP) was used to test tight chain SNPs for conducting MAS and the candidate genes screened across significant genome-wide (GW) QTL localization intervals were further analysed by qRT-PCR. Our results will facilitate the elucidation of the molecular mechanisms that determine growth kinetics, sex determination and hypoxia tolerance-related traits. They will also be useful in future MAS and de novo GSA in *P. vachelli* and in CGA in Siluriformes fish. We provide a good case study demonstrating the utility of genomic data for investigating the genetic basis of important phenotypic traits using reduced-representation sequencing.

## Results

### Characteristics of several phenotypic traits

The mapping family in this study consisted of 200 *P. vachelli* progeny and their phenotypic growth (e.g., W, TL, HL, BL/HL, BH, BL, BW/ED, BL/SL) and hypoxia tolerance-related traits were in concordance with the normal distribution (Kolmogorov-Smirnov test, asymptotic significance > 0.05; Table S1). All growth-related trait values are shown in Table S1, as well as their phenotypic sexes and time to balance loss.

With regards to the growth-related traits, BL/HL, CF, BW/ED, BH/ED, and BL/SL were related to body type proportions and W, BL, HL, TL, BW, BH, CL, and CH were directly related to weight, with these eight traits showing a strong correlation with each other (*r* = 0.593–0.967, *P* < 0.001 for all) (Table 1). The highest correlation value (*r* = 0.967) was observed between BL and TL. There was a strong correlation between W, TL and BL, with the W being strongly correlated with TL (*r* = 0.927) and BL (*r* = 0.934). HL was highly correlated with TL (*r* = 0.813), BL (r = 0.813) and W (*r* = 0.823). By phenotype sexing of the mapping family, 101 and 99 individuals were identified as males and females, respectively, with a sex ratio of 1.02:1.

**Table 1:** Pearson correlation coefficients (r) for all pairwise combinations of the eight weight-related traits (*P* < 0.001 for all)

Traits	BL	BH	W	BW	HL	CH	CL	TL
**BL**	1							
**BH**	0.643	1						
**W**	**0.934**	0.735	1					
**BW**	0.683	0.696	0.736	1				
**HL**	0.813	0.627	0.823	0.667	1			
**CH**	0.697	0.643	0.743	0.677	0.671	1		
**CL**	0.778	0.593	0.731	0.636	0.717	0.643	1	
**TL**	**0.967**	0.664	**0.927**	0.707	0.813	0.702	0.780	1

### Construction and sequencing of ddRAD libraries

A total of 202 ddRAD libraries were constructed from the two parents and their 200 offspring and sequenced on the Illumina HiSeqXten platform to generate 1246.60 million clean reads, comprising approximately 361.68 Gb of sequencing data. The female and male parental data sets contained 7.51 million filtered reads (comprising 2.18 Gb of data with a GC% of 40.67) and 6.39 million filtered reads (comprising 1.85 Gb of data with a GC% of 40.74), respectively. From the 200 offspring, 1232.70 million filtered reads (average of 6.16 million) corresponding to 357.65 Gb of data (average of 1788.24 Mb; ranging from 619.49 to 4969.84 Mb) were produced for SNP detection (Table S2).

### SNP discovery and genotyping

A total of 461,909 raw polymorphic markers were detected using the STACKS pipeline. After stringent selection, 5165 polymorphic markers were successfully genotyped in both parents and offspring. These SNPs were classified into three categories: maternal heterozygous (1881 SNPs), paternal heterozygous (2405 SNPs), and heterozygous in both (879 SNPs). All SNPs are listed in Table S3.

### High-resolution genetic map construction

**Fig. 1:**
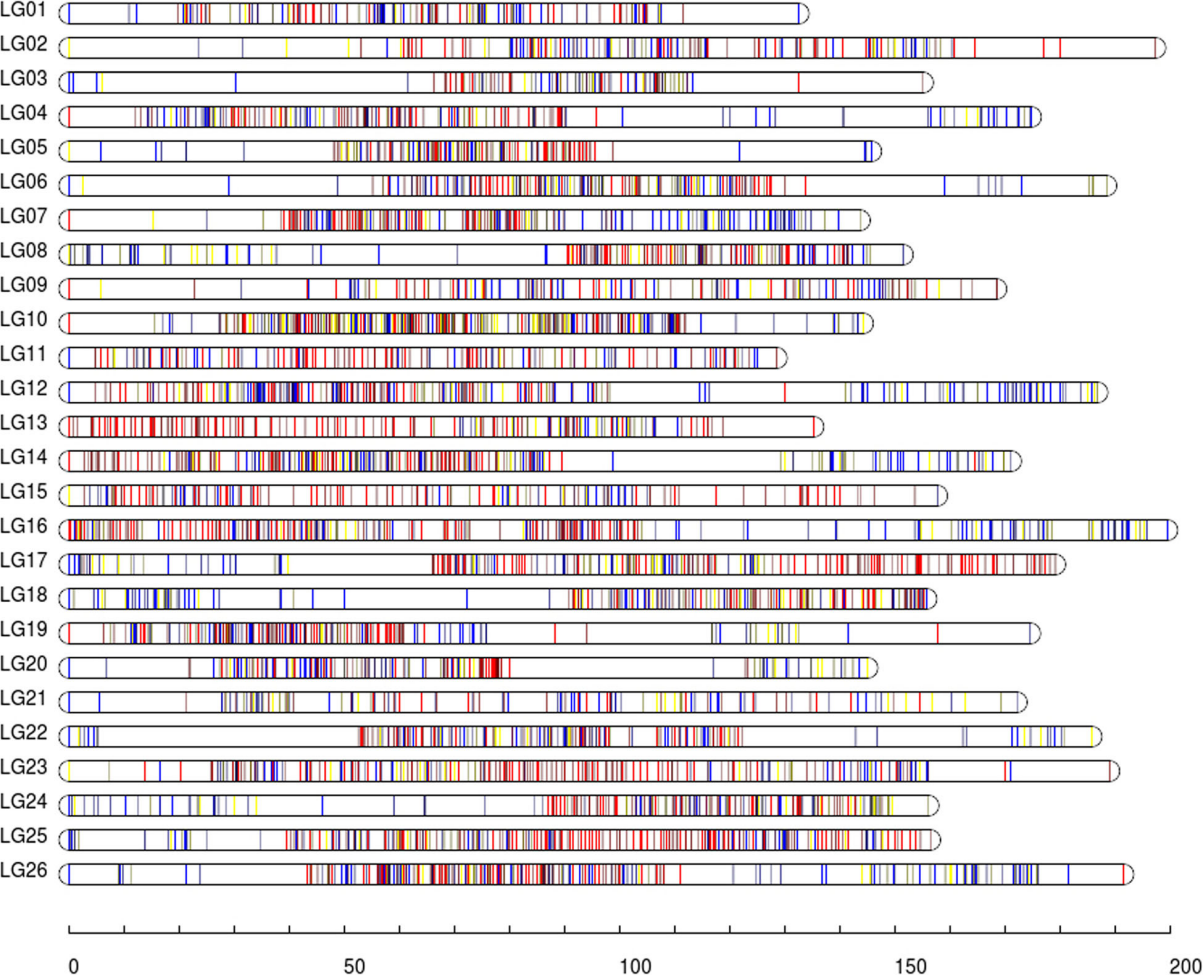
Linkage group lengths and marker distributions of the high-resolution ddRAD-based SNP linkage map of *Pelteobagrus vachelli*. Within each linkage group, red, blue, and yellow lines represent paternal heterozygous SNPs, maternal heterozygous SNPs, and SNPs heterozygous in both parents, respectively. The details of the genetic map are given in Table S4.

A high-resolution ddRAD-based linkage map of *P. vachelli* was constructed using a pseudo-testcross strategy (a mapping population is developed by hybridizing two unrelated, highly heterozygous parents to produce a set of F1 progeny). The resulting integrated map consisted of 26 linkage groups (LGs), including 5059 segregating SNPs (97.9% of all 5165 polymorphic markers). The number of LGs was congruent with the number of *P. vachelli* chromosomes (2n = 52) [33] The total map length was 4047.01 cM with an average interlocus distance of 0.11 cM. The genetic length of each LG ranged from 122.47 cM (LG1) to 189.03 cM (LG24) with an average interlocus distance of 0.03–0.83 cM (Table 2 and Fig. 1). The locus names and SNP positions in the 26 LGs of the integrated genetic map are listed in Table S4.

**Table 2:** Characteristics of the *Pelteobagrus vachelli* genetic maps

LG ID	No. of SNPs	Distance (cM)	Average interlocus distance (cM)	No. of Gaps	Max Gap (cM)
**1**	149	122.47	0.83	3	19.50
**2**	149	163.94	0.07	2	14.39
**3**	106	178.22	0.04	5	25.97
**4**	177	167.48	0.04	4	17.54
**5**	149	127.38	0.03	3	20.16
**6**	189	139.43	0.03	3	18.65
**7**	209	166.37	0.04	1	11.90
**8**	172	178.06	0.04	4	18.76
**9**	148	175.51	0.03	2	7.55
**10**	368	167.40	0.32	5	7.88
**11**	152	136.36	0.2	0	3.72
**12**	258	152.45	0.16	3	13.36
**13**	161	127.96	0.12	1	15.51
**14**	247	147.11	0.11	2	26.11
**15**	147	124.62	0.08	2	5.76
**16**	262	170.70	0.1	4	9.30
**17**	228	175.63	0.09	1	25.67
**18**	181	140.69	0.07	3	20.13
**19**	206	178.45	0.08	6	23.25
**20**	159	133.43	0.05	2	33.97
**21**	122	172.85	0.06	3	6.93
**22**	179	136.32	0.05	4	15.06
**23**	245	151.60	0.05	2	14.41
**24**	184	189.03	0.06	6	15.78
**25**	254	137.01	0.04	0	2.57
**26**	258	186.54	0.05	6	19.11

### Comparative genome mapping

**Fig. 2:**
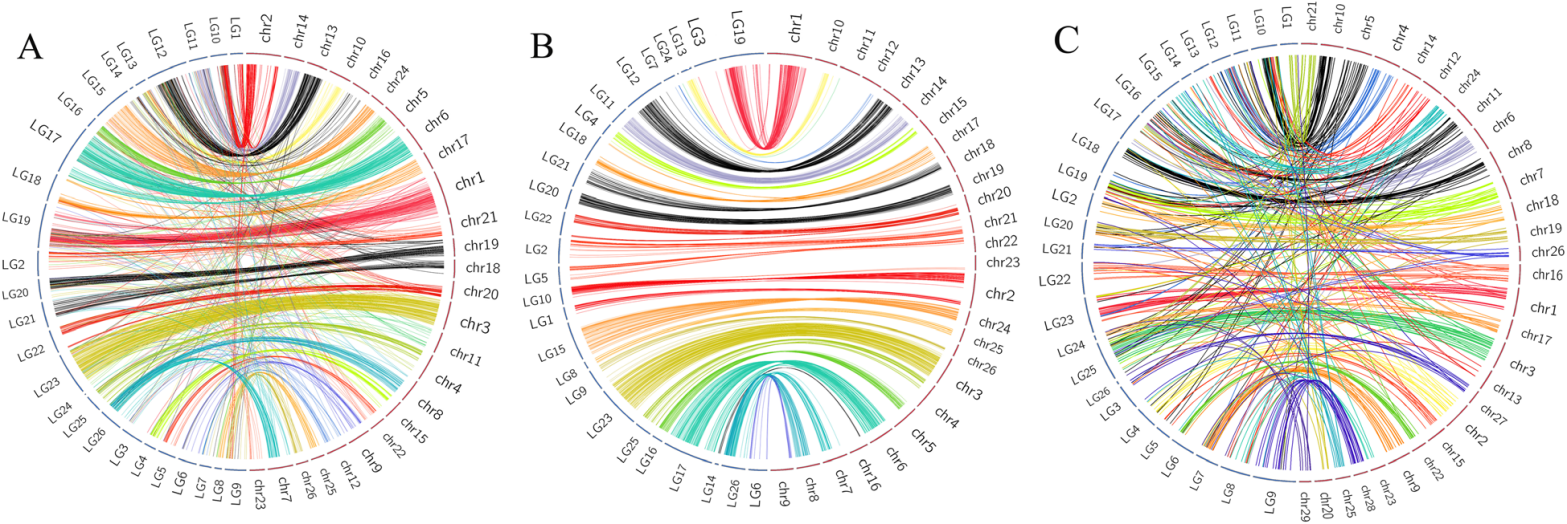
Circos diagrams representing syntenic relationships between *P. vachelli* and *P. fulvidraco* (**a** and **b**) and *I. punctatus* (**c**). Only markers of each linkage group of *P. vachelli* that were mapped to a single chromosome of *P. fulvidraco* are shown in B

Within the comparative genome mapping between the LGs of *P. vachelli* and the chromosomes of *P. fulvidraco*, 3879 (76.7% of 5059) markers were uniquely mapped to the assembled LGs and were used for synteny analysis (Fig. 2a). Overall, a one-to-one correspondence was observed between the LGs of *P. vachelli* and the chromosomes of *P. fulvidraco*. Notably, for each LG of *P. vachelli*, approximately 83.2% of the 3879 synteny markers were located on a single chromosome of *P. fulvidraco* (Fig. 2b), while the remaining were dispersed into various LGs (Table S5). A total of 871 (17.2% of 5059) markers in the linkage map of *P. vachelli* were uniquely aligned to the chromosomes of *I. punctatus* (Table S5 and Fig. 2c).

### QTL mapping of significant economic traits

**Fig. 3:**
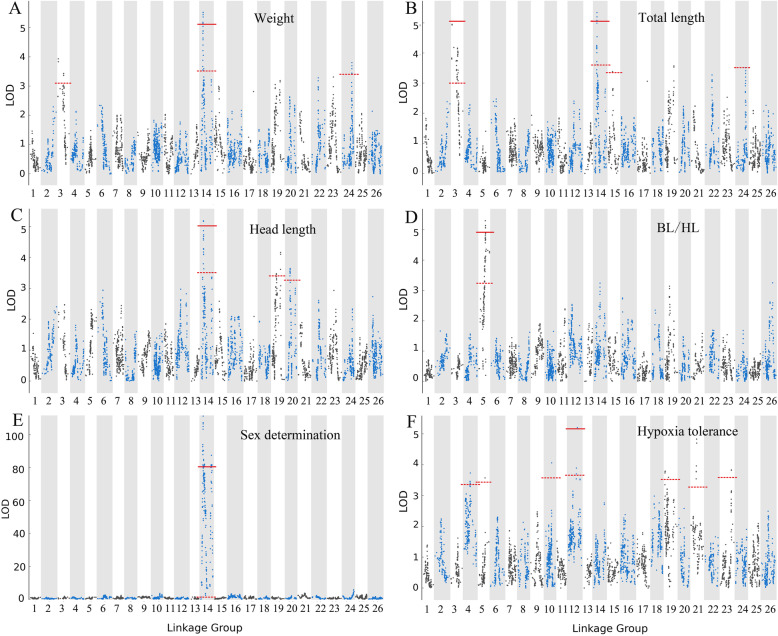
LOD scores along the 26 linkage groups for the variations in the 8 significant genome-wide QTLs associated with phenotypic traits (**a**. weight, **b**. total length, **c**. head length, **d**. BL/HL, E. sex determination, F. hypoxia tolerance) in *Pelteobagrus vachelli*. LOD significance threshold levels were determined on the basis of 1000 permutations at a significance level of *P* < 0.05. The dashed and solid lines indicate the chromosome-wide (CW) and genome-wide (GW) significance thresholds, respectively

In this study, we conducted 15 QTL location analyses, including 13 growth (5 body proportion- and 8 weight-related traits), sex determination-, and hypoxia tolerance-related traits. For body proportion-related traits, the CW and GW LOD significance thresholds varied from 3.1 to 3.3 and from 4.9 to 5.7, respectively, based on the permutation test. Using the interval mapping algorithm, no significant QTLs were associated with BW/ED. A total of 11 QTLs that associated with weight-related traits were detected in 6 LGs, including 1 significant GW QTL (qBL/HL5-a; Fig. 3d) and 10 significant CW QTLs, with LOD scores ranging from 3.1 to 5.1 (Table 3).

**Table 3:** Detected QTLs associated with body proportion-related traits in *Pelteobagrus vachelli*

Body proportion-related traits	QTL	LG	CI (cM)	LOD	GW	CW	PVE
**BL/HL**	**qBL/HL5-a**/b/c	**5**	**79.09–92.41**/74.52 −77.10/93.64–145.70	**5.3**/3.8/4.36	**4.9**	3.2	**11.5**/8.4/9.5
**Condition factor**	qCF9-a/b/c	9	55.75–55.75/65.33 −65.33/69.72–75.22	3.2/3.1/3.51	5.4	3.1	7.1/6.9/7.8
	qCF11-a	11	51.74–51.74	3.32		3.2	7.4
	qCF24-a	24	96.94–96.94	3.91		3.3	8.6
**BH/ED**	qBH/ED15-a/b	15	64.78–65.90/78.00–78.03	3.4/3.36	5.7	3.3	7.5/7.4
**BL/SL**	qBL/SL2-a	2	105.65–107.97	3.52	5.1	3.2	7.8

*LG* Linkage group, *CI* Confidence interval, *GW* Genome-wide, *CW* chromosome-wide, *PVE* phenotypic variance explained. The numbers in bold represent significant GW QTLs

For weight-related traits, the CW and GW LOD significance thresholds varied from 3 to 3.6 and from 5 to 5.1, respectively, based on the permutation test. A total of 36 QTLs associated with weight-related traits were detected in 10 LGs using the interval mapping algorithm, including 4 significant GW QTLs (qW14-a, qTL3-a, qTL14-a and qHL14-a; Fig. 3a, b, c) and 19 significant CW QTLs, with LOD scores ranging from 3.26 to 5.52 (Table 4). Five QTLs (qW14-a/b, qW24-a and qW3-a/b) associated with W were located at 38.938 cM, 48.111 cM, 129.243 cM, 5.016 cM, and 80.194 cM along LG3, LG14 and LG24 and contributed phenotypic variance explained (PVE) values of 12, 7.9, 8.4, 8.6 and 7.6%, respectively (Table 4 and Fig. 3a). Owing to the strong correlation between W, TL and BL, QTLs for BL and TL were located within the same confidence intervals along three LGs (Table 4). Six QTLs for HL were mapped to LG14, LG19 and LG20 with PVE values ranging from 7.2 to 11.2%. Two QTLs associated with BW were located within LG2 and LG6 with PVE values of 7.4 and 7.7%, respectively. For CH, five QTLs located within LG14 and LG22 contributed PVE values of 7.3–9%, with LOD scores of 3.3–4.09. Four QTLs for CL were mapped to LG3, LG19 and LG23 with PVE values ranging from 7.5–9.4%. Two QTLs associated with BH were located within LG15 with PVE values of 7.4 and 7.5% (Fig. 3d). Among all QTLs, a total of 18 confidence intervals within LG3 and LG14 were associated with weight-related traits and in addition, all significant GW QTLs were mapped to these two LGs (Table 4).

**Table 4:** Detected QTLs associated with weight-related traits in *Pelteobagrus vachelli*

Weight-related traits	QTL	LG	CI (cM)	LOD	GW	CW	PVE
**Weight**	**qW14-a**/b	**14**	**34.47–41.73**/48.11–48.11	**5.52**/3.59	**5.1**	3.5	**12**/7.9
	qW24-a	24	128.69–130.23	3.8		3.3	8.4
	qW3-a/b	3	5.02–6.02/79.99–80.37	3.93/3.43		3.1	8.6/7.6
**Total length**	**qTL3-a**/b	**3**	**0–70.33**/75.64–88.55	**5.14**/4.22	**5.1**	3	**11.2**/9.3
	**qTL14-a**/b	**14**	**37.01–41.73**/36.78–36.78	**5.37**/4.25		3.6	**11.6**/9.3
	qTL15-a	15	60.39–60.39	3.38		3.2	7.5
	qTL24-a/b	24	125.89–125.89/128.91–129.24	3.51/3.51		3.5	7.8/7.8
**Body length**	qBL3-a/b	3	0–69.24/78.1–88.55	4.67/4.16	5	3.1	10.2/9.1
	qBL14-a	14	36.15–40.91	4.87		3.5	10.6
	qBL24-a/b	24	125.89–125.89/129.24–129.24	3.42/3.3		3.3	7.6/7.3
**Head length**	**qHL14-a**/b	**14**	**35.29–40.91**/156.21–156.21	**5.18**/3.51	**5**	3.5	**11.2**/7.8
	qHL19-a/b	19	63.30–64.51/122.93–123.84	3.49/4.16		3.4	7.7/9.1
	qHL20-a/b	20	51.39–51.39/53.70–56.85	3.26/3.7		3.2	7.2/8.2
**Body width**	qBW2-a	2	197.32–197.32	3.36	5	3.3	7.4
	qBW6-a	6	57.10–58.28	3.48		3.3	7.7
**Caudal peduncle height**	qCH14-a/b/c/d	14	36.15–36.78/37.10–37.42/ 38.94–41.73/48.11–48.11	4.09/3.79/ 3.97/3.48	5.1	3.5	9/8.3/ 8.7/7.7
	qCH22-a	22	176.48–176.48	3.3		3.3	7.3
**Caudal peduncle length**	qCL3-a	3	5.02–30.27	4.28	5.1	3	9.4
	qCL19-a	19	123.67–123.84	3.48		3.3	7.7
	qCL23-a/b	23	43.08–43.08/85.87–85.87	3.4/3.45		3.4	7.5/7.6
**Body height**	qBH15-a/b	15	20.29–20.68/26.42–26.42	3.36/3.4	5	3.3	7.4/7.5

*LG* Linkage group, *CI* Confidence interval, *GW* Genome-wide, *CW* Chromosome-wide, *PVE* Phenotypic variance explained. The numbers in bold represent significant GW QTLs

For the detection of sex determination loci, the CW and GW LOD significance thresholds varied from 3.3–3.6 and 80.4, respectively, based on the permutation test. A total of 6 QTLs associated with sex-related traits were detected within 3 LGs, including 2 significant GW QTLs (qSXE14-a/b) and 4 significant CW QTLs, with LOD scores ranging from 3.46 to 111.33 (Fig. 3e and Table 5).

**Table 5:** Detected QTLs associated with sex determination- and hypoxia tolerance-related traits in *Pelteobagrus vachelli*

Traits	QTL	LG	CI (cM)	LOD	GW	CW	PVE
**Sex determination**	**qSXE14-a/b**	**14**	**11.36–68.23/69.24–171.10**	**111.33/87.49**	**84.4**	3.6	**92.3/86.7**
	qSXE21-a/b	21	90.12–92.73/98.26–98.38	3.64/3.46		3.3	8/7.6
	qSXE24-a/b	24	145.86–145.86/146.88–156.10	3.48/5.69		3.4	7.7/12.3
**Hypoxia tolerance**	**qHT12-a**	**12**	**115.51–129.99**	**5.22**	**5.1**	**3.7**	**11.3**
	qHT4-a/b/c/d	4	81.61–81.61/84.14–84.66/	3.34/3.46/		3.3	7.4/7.7/
			87.39–87.39/88.72–88.72	3.39/3.73			7.5/8.2
	qHT5-a	5	93.28–93.28	3.57		3.4	7.9
	qHT10-a	10	95.71–95.71	4.06		3.6	8.9
	qHT19-a/b	19	8.13–11.29/57.51–57.51	3.79/3.59		3.5	8.3/7.9
	qHT21-a	21	72.53–79.79	4.83		3.2	10.5
	qHT23-a	23	153.79–153.82	3.83		3.6	8.4

*LG* Linkage group, *CI* Confidence interval, *GW* Genome-wide, *CW* Chromosome-wide, *PVE* Phenotypic variance explained. The numbers in bold represent the significant GW QTLs

For hypoxia tolerance-related traits, the CW and GW LOD significance thresholds varied from 3.2–3.7 and 5.1, respectively, based on the permutation test. A total of 11 QTLs associated with hypoxia tolerance-related traits were detected within 7 LGs using the interval mapping algorithm, including 1 significant GW QTL (qHT12-a) and 10 significant CW QTLs, with LOD scores ranging from 3.34 to 5.22 (Fig. 3f and Table 5).

### SNP association with significant traits

To confirm and refine the position of these significant GW QTLs, the 200 remaining individuals and the two parents of the 400 offspring, as well as 48 independent wild individuals (24 ♀:24 ♂) were used for SNP phenotypic association analysis by Kompetitive Allele Specific PCR (KASP) assays (Table S6). Our results showed that growth-related traits, including body proportion (BL/HL; un_16957051) and weight (W, BL, TL, HL, and CH) were closely related to several markers (un_15322259, un_40276574, un_36488182, un_47032303, un_34909841, and un_54585281) with a K* of 3.884–9.534 and a significance of 0.002–0.049. The un_28380227 marker showed the highest association with the hypoxia locus from 1 significant GW QTL (qHT12-a) with a K* of 4.72 and a significance of 0.03.

**Fig. 4:**
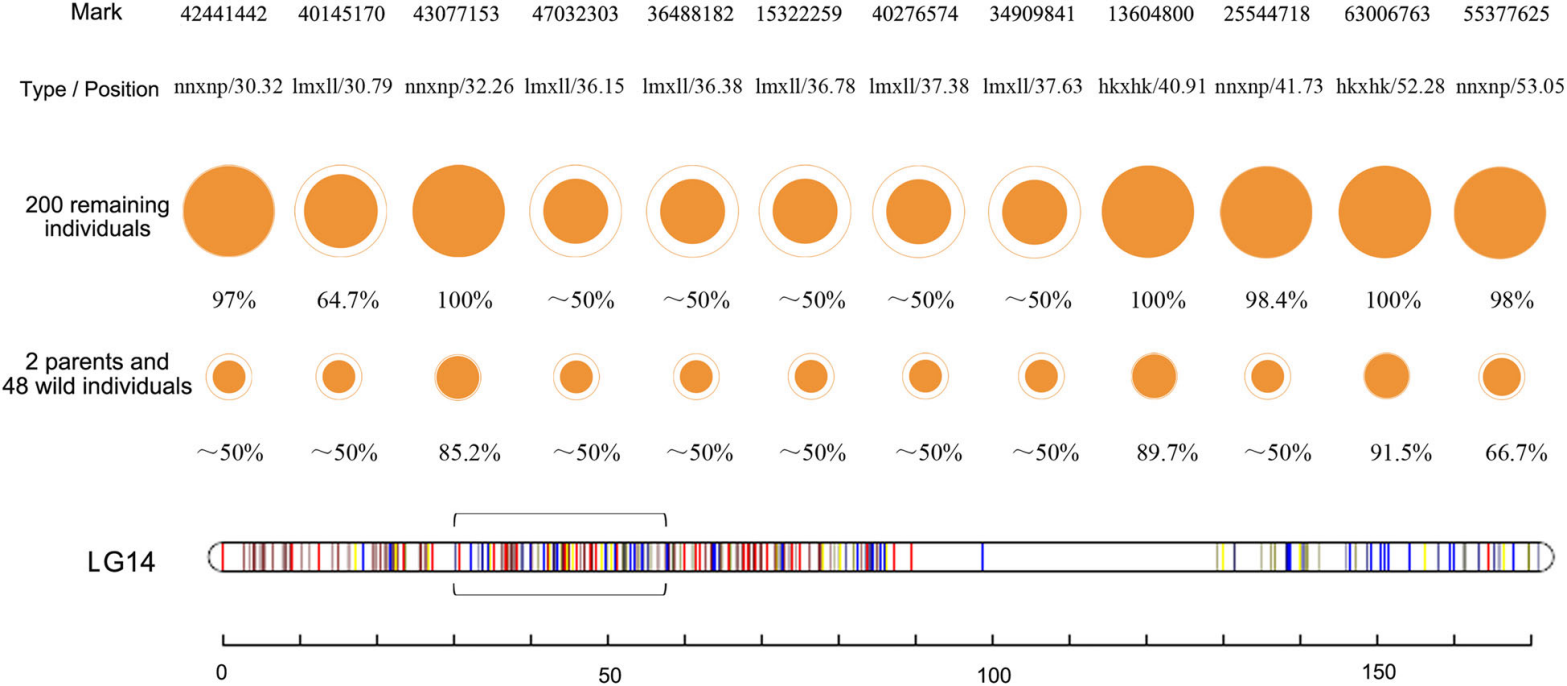
KASP assays of SNP sex association. Details for the 12 markers tested by KASP assays for the significant GW QTLs of sex determination. From top to bottom: 12 marker IDs; SNP type and SNP position in the genetic map in cM; KASP assays results of 200 remaining individuals and 2 parents and 46 wild individuals. The outer circle diameters for the KASP assay results are proportional to the number of alleles successfully tested; the inner disks represent the genotype of the markers consistent with phenotypic sex based on the ddRAD-Seq; percentage = the inner disk / the outer circle. Detailed data are provided in Table S6. nnxnp: homozygous ♂ and heterozygous ♀; hkxhk: heterozygous ♂ and heterozygous ♀; lmxll: homozygous ♀ and heterozygous ♂

All of the genotypic types (hkxhk, lmxll, and nnxnp) of the 200 remaining individuals were verified and in accordance with their parents. For these 12 sex-associated SNPs, the loci with hkxhk and nnxnp were found to be completely related to the gender phenotype in the 200 remaining individuals (over 97%). For example, in each case the remaining individuals were homozygous (nn) male and heterozygous (np) female when the parents were a homozygous ♂ and a heterozygous ♀ (nnxnp) for these markers. If both parents were heterozygous (hkxhk), the offspring were homozygous with one exclusively male (hh) and the other exclusively female (kk), and the heterozygous offspring (hk) were either male or female. Sex-associated SNPs could not be determined with the lmxll marker (approximately 50–64.7%; Fig. 4 and Table S6). Most loci in the wild population showed no gender difference (approximately 50%), but there were several potentially valuable loci to consider as a marker for potential gender determination, with up to 85.2% association (un_43077153, un_13604800, and un_63006763).

### Potential candidate genes for the 8 significant GW QTLs

**Fig. 5:**
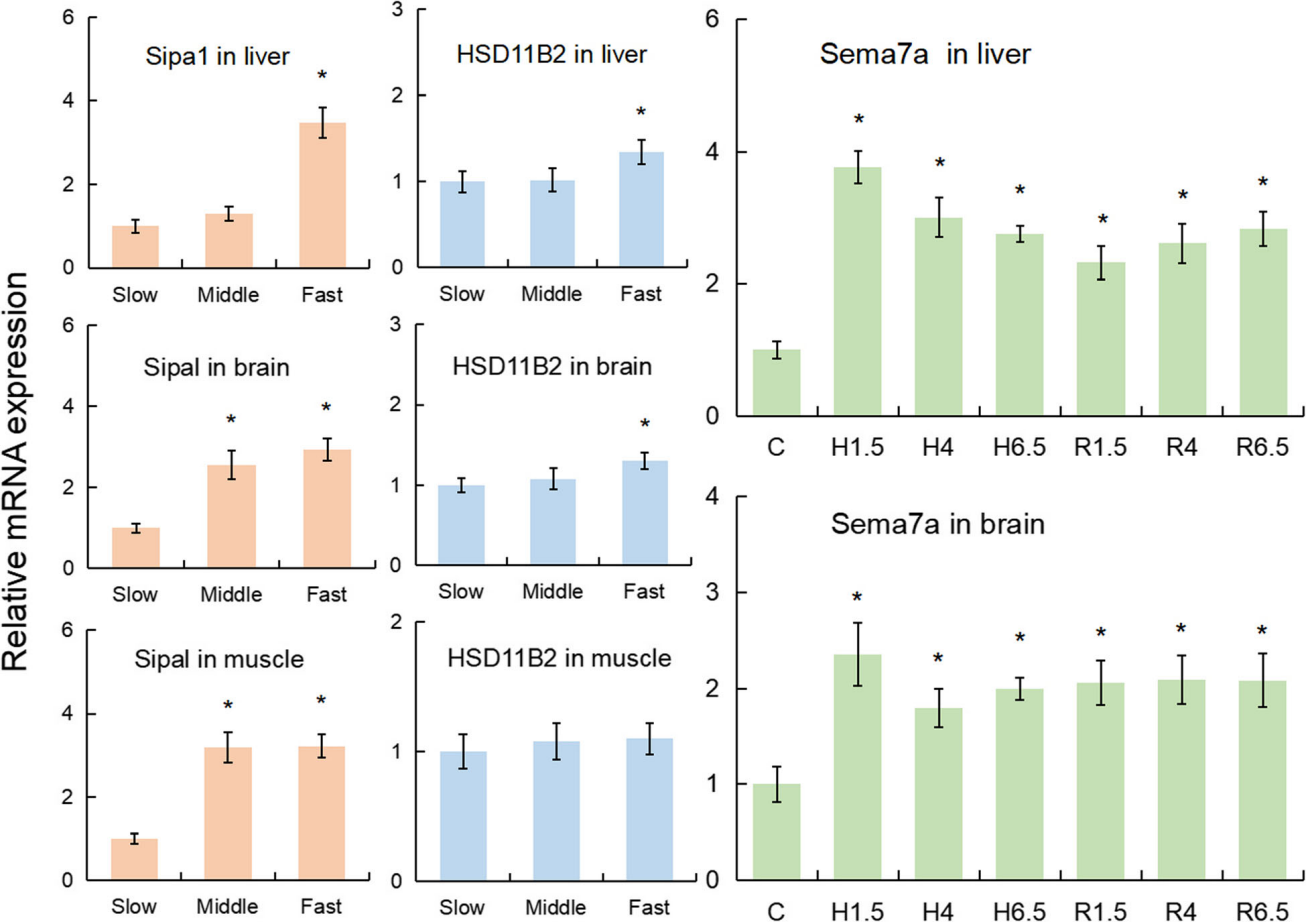
Left side: The expression pattern of Signal-induced proliferation-associated 1-like protein 1 (Sipa1) and Corticosteroid 11-beta-dehydrogenase isozyme 2 (HSD11B2) in slow- medium- and fast- growing fish. Right side: The temporal expression profiles of Semaphorin-7A (Sema-7a) mRNA in the liver and brain after acute hypoxia and reoxygenation, as determined by qRT-PCR. Statistical analyses of differences were conducted by one-way analysis of variance (ANOVA) using SPSS 22.0 software. Error bars represent the standard deviation of three replicates. The asterisks above the bars represents statistically significant differences from control fish (*P < 0.05). Control, hypoxia (1.5, 4 and 6.5 h) and reoxygenation (1.5, 4 and 6.5 h) values are shown as bars C, H1.5, H4, H6.5, R1.5, R4 and R6.5, respectively

Based on the non-redundant annotation information of the *P. fulvidraco* genome, 72 genes in total were located within the 8 significant GW QTLs (4 weight, 1 body proportion, 2 sex determination, and 1 hypoxia tolerance; Table S7). Among them, 2 genes from 1 body proportion-related QTL (qBL/HL5-a) are related to minus end-directed microtubule transport and sphingolipid synthesis. A total of 14 genes from 4 weight-related QTLs (qW14-a, qTL3-a, qTL14-a, qHL14-a) play important roles in the genetic regulation of development, cell proliferation, immunity, and energy metabolism. The expression of the rapid growth-related genes, Signal-induced proliferation-associated 1-like protein 1 (Sipa1) and Corticosteroid 11-beta-dehydrogenase isozyme 2 (HSD11B2), was significantly higher in fast-growing fish than in slow-growing fish in both the liver and the brain (*P* < 0.05, two-tailed t-test) and Sipa1 expression in the muscle of slow-growing fish was significantly lower than in fast- and medium-growth fish (Fig. 5).

A total of 55 candidate QTL genes from 2 sex-related QTLs (qSXE14-a/b) were identified, however, no genes associated with sexual differentiation or determination were identified within this region based on the non-redundant annotation information of the *P. fulvidraco* genome (Table S7). The gene Semaphorin-7A (Sema7a) from 1 of the hypoxia tolerance-related traits (qHT12-a) has been reported to play an important role in hypoxia adaption (Table S7). The temporal expression of Sema-7a mRNA changed in the liver and brain after hypoxia and reoxygenation. Sema-7a mRNA expression peaked in both the liver and the brain after 1.5 h of hypoxia and remained high throughout the recovery.

## Discussion

### High-resolution genetic map construction using ddRAD-based SNPs

To date, most catfish genetic maps have been constructed using expressed sequence tags [34], AFLP [35], and SSR [36]. The density of these linkage maps in catfish is still low, with an average density of 1.40–15.20 cM. Currently, there have been several attempts to construct high-density genetic maps of catfish, but thus far, only channel catfish (*I. punctatus*) [37] and southern catfish (*Silurus meridionalis*) [38] maps have been constructed. No high-density linkage maps for any Bagridae catfish species have been generated. In the present study, a high-resolution genetic linkage map of dark barbell catfish (*P. vachelli*) was constructed with 5059 SNPs using the ddRAD-seq technique, which represents the most saturated linkage map to date. This is the first high-density Bagridae fish genetic map reported. A high-density linkage map, such as the one we have constructed, provides detailed information on genomic structure and is a valuable resource for comparative genomics and fine-scale QTL mapping in catfish.

### Comparative genome analysis

Comparative mapping with model species is an efficient way to evaluate the accuracy of genetic maps of non-model species. In this research, syntenic analyses were performed between our map and the reference genome of *P. fulvidraco,* a species belonging to the same genus as *P. vachelli*. The number of LGs was congruent with the number of *P. fulvidraco* chromosomes (2n = 52) [18]. A near 1:1 relationship was observed between our map and the draft genome of *P. fulvidraco* and a high level of conserved genomic synteny (76.7%) and syntenic boxes (83.2%) were presented, which indicates a high genetic relationship between *P. vachelli* and *P. fulvidraco*. A similar phenomenon was also detected for the previous linkage map of *Cyprinus carpio* [39]. However, only 17.2% of the mapped markers were uniquely aligned to the chromosomes of *I. punctatus* (2n = 58), indicating the presence of extensive intra-chromosomal rearrangements between the two catfish species or issues with the assembly of the draft genome [40].

### Fine scale QTL mapping and SNPs phenotypic association

Fine scale QTL mapping is an efficient approach to identify genetic loci and candidate genes underlying quantitative traits of interest. To date, growth-related QTL studies have been reported in various aquatic species, but with the exception of those in *I. punctatus* [26, 27], very few studies including fine scale QTL mapping have been performed in catfish. QTL fine mapping and candidate gene identification were difficult to achieve in *P. vachelli* due to the absence of a high-density linkage map. Within this study, our map contains abundant high confidence QTLs related to weight, body proportion, sex determination and hypoxia tolerance. These findings represent a promising platform for MAS and GAS in catfish and will help to clarify the molecular mechanisms of the above phenotypic traits.

Validating the results of the QTL association analysis is of the utmost importance. The fact that the phenotype-associated SNPs showed strong association with weight, body proportion (BL/HL), sex determination, and hypoxia tolerance traits when tested in the 200 remaining individuals, two parents and 48 independent wild individuals using KASP assays is promising. Interestingly, we found that several markers were associated with multiple traits (W, BL, TL, HL, CH) at the same time (Table 6), which also reflects the strong correlation between these weight-related traits. For the 12 sex-associated SNPs, several loci, including hkxhk and nnxnp, showed great relevance to the gender phenotypes (over 97% association in the remaining individuals and over 85.2% association in the wild population), but most loci showed no gender differences (approximately 50% association) in the wild population. The above results also demonstrate the limitations of using SNPs as a sex marker, because they are susceptible to the influence of the parents within the family, which has also been observed in sex association analyses of *O. niloticus* L. [17], *H. hippoglossus* [16], and *P. oxygeneios* [32]. Nonetheless, the use of these markers is possible to conduct genetic gender identification and all-male breeding based on the *P. vachelli* family line selection. MAS could also be conducted using these SNPs, providing a valuable tool towards the more efficient production of all-male, fast growing and hypoxia tolerant *P. vachelli* for the aquaculture industry.

**Table 6:** Details of the MAS markers tested by KASP assays for the significant GW QTLs of growth and hypoxia tolerance-related traits

Marker	Type	LG	Trait	LOD	K*	Significance
**un_16957051**	hkxhk	5	BL/HL	5.03	8.895	0.012
**un_15322259**	lmxll	14	W, BL, TL, HL	4.25–5.18	5.094–8.397	0.004–0.024
**un_40276574**	lmxll	14	W, BL, TL, HL, CH	3.77–5.04	4.901–8.682	0.003–0.027
**un_36488182**	lmxll	14	W, BL, TL, HL, CH	3.44–4.88	4.726–9.534	0.002–0.03
**un_47032303**	lmxll	14	W, BL, TL, HL	3.67–4.8	5.836–8.581	0.003–0.016
**un_34909841**	lmxll	14	W, BL, TL, HL, CH	3.04–4.4	3.884–8.023	0.005–0.049
**un_54585281**	hkxhk	3	TL, BL	4.57–4.93	7.458–7.954	0.019–0.024
**un_28380227**	lmxll	12	Hypoxia tolerance	5.22	4.72	0.03

K*: Kruskal-Wallis test statistic; significance: asymptotic significance level; pos: SNP position in LG. Detailed data are provided in Table S6. nnxnp: homozygous ♂ and heterozygous ♀; hkxhk: heterozygous ♂ and heterozygous ♀; lmxll: homozygous ♀ and heterozygous ♂

### Growth-related traits and candidate genes

Strong correlations among the growth-related traits (BW, TL, BL, BH and HL) have been reported in some aquaculture species [39]. Similarly, these five growth-related traits were also highly correlated with each other in this study (Table 1). As expected, based on the relatively high correlation value (mean *r* = 0.943) between W, TL and BL, QTLs for W, TL and BL had the same distribution patterns within the three LGs (LG3, LG14, and LG24). Additionally, all the significant GW QTLs associated with weight-related traits were detected within LG3 and LG14, indicating that these two major LGs are involved in rapid growth and that the candidate genes associated with weight may also be located within these LGs. Among the significant GW QTLs, it is worth noting that several genes related to T cell development (thymocyte selection-associated high mobility group box protein, TOX), T cell antigen recognition (T cell receptor beta variable region), and the inducible expression of T cell cytokine genes (nuclear factor of activated T cells, cytoplasmic 3) were identified, indicating that strong disease resistance may contribute to individual growth [41, 42].

In addition, two genes, Sipa1 and HSD11B2, were the most likely to be associated with growth, given their important roles in cell proliferation and development. Sipa1 stimulates the GTPase activity of the Ras-related protein Rap-2a, which may well regulate cytoskeletal rearrangements, cell migration, cell adhesion and cell spreading [43, 44], as well as promote the reorganization of the actin cytoskeleton and the recruitment of disks large homologue 4 to F-actin [45]. In mammals, glucocorticoid is very decisive in cell proliferation and differentiation, with the two glucocorticoid metabolic enzymes, HSD11B1 and HSD11B2, considered as anti-proliferation and pro-proliferation switches in cells, respectively. During embryonic development, HSD11B2 prevents cells from the growth-inhibiting and/or pro-apoptotic effects of cortisol [46]. In fish, HSD11B2 was considered to have an activity that is similar to that of mammalian HSD11B2 in preventing the cortisol activation of the glucocorticoid receptors, which could mediate cell development and stress responses. Additionally, HSD11B2 catalyses 11b-hydroxytestosterone converses to 11-keto-testosterone, a fish androgen. Previous studies have reported that androgen-related genes (e.g., 17α-Methyltestosterone) could improve the GH/IGF axis and growth in *P. fulvidraco* [47]. It was also found that HSD11B2 increases gradually during the period of individual growth in two catfish species [48, 49]. Interestingly, the expression of Sipa1 and HSD11B2 in both the liver and brain of the fast-growing fish was significantly higher than that in the medium- or slow-growth fish. This is consistent with the function of these genes in cell proliferation and development, supporting the notion that Sipa1 and HSD11B2 are the two good candidate genes for growth. However, with the current data, we cannot rule out the involvement of other genes within the significant QTLs for growth and further detailed studies on gene function are required to understand the underlying mechanisms of differential growth among individuals.

### Sex determination-related traits and candidate genes

The male *P. vachelli* exhibit > 30% faster growth than females and therefore, in both the *P. vachelli* farming and academic fields, the development of all-male *P. vachelli* for aquaculture has been a topic of great concern. The sex determination of *P. vachelli* is XX/XY because sex ratios in conventional diploid offspring approximate 1:1 [19, 33] and gynogenetic offspring are all female (unpublished data), which is the same in *P. fulvidraco*. However, similar to other fish species, it is not easy to distinguish between the sex chromosomes (X and Y), as well as between the sex and autosomal chromosomes based on current cytogenetic techniques. Screening of sex-associated markers will make the time required for all-male *P* to develop much shorter [50]. *vachelli*. In this study, two significant GW QTLs for sex determination in *P. vachelli* were identified on LG14 (with 92.3% of PVE). There were also some significant CW QTLs within LG21 and LG24, which is very similar to *S. maximus*. The most sex-related QTLs in *S. maximus* are found within LG21 (with 99.9% of PVE), with a linkage span of 70.882 cM. In LG7 and LG14, a small quantity of QTLs with high feasibility are discovered too [4]. In some other fish species, the similar phenomena have also been found. In *H. hippoglossus*, four markers which can be located in LG13 were found to be related to sex determination significantly, with 82% of PVE associated with sex [16]. Additionally, in *Sparus aurata* L. [3] and *S. chuatsi* [51], the significant QTLs affecting sex determination were found to be located in LG21 and LG23, respectively. It is suggested that, in some fish species, a main LG or chromosome is involved in sex determination and that in this LG, the sex determination genes may be located.

Among the significant GW QTLs of sex determination, not a single gene associated with sexual differentiation or determination was identified in this area, which may be due to the limitations of our simplified genome sequencing data or to the limitation that the *P. fulvidraco* genome came from an XX individual. Among the candidate genes, thrombospondin-3b was the most likely to be associated with gender development. Thrombospondin (TSP) proteins are a major component of cortical rods in mature oocytes in invertebrates [52]. A previous study has reported that two types of TSPs from *O. niloticus* involved in the formation of gonads and oogenesis also play an important role in the 14 day spawning cycle [53].

### Hypoxia tolerant traits and candidate genes

The hypoxia tolerant trait was found to be a quantitative trait that is controlled by many genes in animals [54]. Mapping QTLs for hypoxia traits associated with tolerance is the first step in improving the stress tolerance of breeding fish, but few QTL mapping studies on hypoxia traits in fish have been conducted. Only recently, two genome-wide association studies were carried out to identify QTLs for hypoxia tolerance using 250 K SNP arrays and ddRAD-seq with channel catfish families [26, 27] and *O. niloticus* [28], respectively. It is worth noting that we identified a marker, un_28380227, that could be directly used in the MAS of tolerant lines in *P. vachelli*. Additionally, our findings of the markers located on significant GW QTL intervals in LG12 provide a useful resource. Semaphorin-7A (Sema7a) was located in the significant GW hypoxia QTL. Sema7a plays an important role in integrin-mediated signalling and functions in regulating both cell migration and immune responses. Co-immunoprecipitation assays showed that hypoxia-inducible factor-1 (hif-1) had a structure that interacts with Sema7a and that overexpression of hif-1 induces Sema7a production even in normoxic conditions [55]. Previous reports suggested that hypoxia-induced Sema7a could promote neutrophil migration and a pro-tumourigenic mesenchymal phenotype [55, 56], which also correlated with the severity of inflammation and osteoclastogenesis [57]. Furthermore, Sema7a was highly expressed in the early stages of hypoxic stimulation and remained high in various tissues throughout the recovery period. These results suggest that Sema7a plays a vital role during hypoxia and reoxygenation, indicating that Sema7a may be a good candidate gene for the hypoxic response.

## Conclusion

We constructed a high-resolution genetic linkage map with a length of 4047.01 cM and an average marker interval of 0.11 cM using 5059 ddRAD markers in P. vachelli. Comparative genome mapping revealed that a high proportion (83.2%) of markers with a one-to-one correspondence were observed between *P. vachelli* and *P. fulvidraco*. Several SNPs from 8 significant genome-wide QTLs (4 weight, 1 body proportion, 2 sex, and 1 hypoxia) were found to be associated with these phenotypic traits in other individuals by KASP assays. In addition, two candidate genes for weight, Sipa1 and HSD11B2, were differentially expressed between fast-, medium- and slow-growing *P. vachelli*. Sema7a, which is associated with hypoxia tolerance, was induced after hypoxia exposure and reoxygenation. We mapped a set of suggestive and significant QTLs, as well as candidate genes for growth-, sex determination- and hypoxia tolerance-related trait based on the *P. fulvidraco* genome. Our results provide a valuable tool for more efficient production of all-male, fast growing and hypoxia tolerant *P. vachelli* for the aquaculture industry.

## Methods

### Mapping population and phenotypic data

The male parent was selected from a group of *P. vachelli* derived from a wild population from the Pearl River in Guangdong province and the female parent was chosen from a cultured population from the ChenQiang fish farm in Changzhou, Jiangsu Province, China. Fish breeding and cultivation were performed at the Nanjing Normal University. The juvenile fish were fed live *Artemia nauplii* to satiation twice daily until the mouth gape sizes matched the size of the artificial compound feed. The feed was slightly over-supplied based on previous consumption.

Fish were acclimated at an ambient temperature of 26 ± 1 °C in aerated flow-through water with a bio-filtered water recirculation system for 1 week, followed by feed restriction for 2 days. For phenotypic data collection, a total of 400 offspring from a full-sib family at 105 days post-hatch were randomly selected and stocked in an aquarium (580 L of volume). The dissolved oxygen level was reduced gradually from 8.23 to 1.51 mg/L over 2.8 h by bubbling pure nitrogen gas. After that, the surface of the water was enclosed by a plastic film and fish were monitored for signs of balance loss due to hypoxic stress as the dissolved oxygen level was reduced from 1.51 to 0.32 mg/L over 2.6 h. The time and sequence of balance loss for each fish was recorded for the analysis of hypoxia tolerance.

Fish were stopped breathing after anaesthetization in a eugenol bath (1:10,000) for 5.5 min, then dissection directly. Growth related traits including weight (W), body length (BL), condition factor (CF), head length (HL), BL/HL, total length (TL), body width (BW), body height (BH), BW/ED (eye distance), BH/ED (eye diameter), BL/SL (snout length), caudal peduncle length (CL), and caudal peduncle height (CH) were measured by electronic scales or Vernier calliper for all 400 offspring, at which time the sexes were also identified by the stained squash technique. After the measurements were taken, a portion of the fins and muscle tissues were frozen in liquid nitrogen and stored at − 80 °C. This family exhibited a high variation in their growth- and hypoxia tolerance-related traits and therefore 200 randomly selected *P. vachelli* offspring (F1) from the above 400 offspring and their parents (F0) were used for the development of a genetic linkage map and QTL analysis. The 200 remaining individuals were used to test SNP markers in KASP assays.

### Construction and sequencing of ddRAD libraries

Genomic DNA was extracted from the fin tissue of each of the 200 randomly selected offspring (F1) and two parents (F0). ddRAD libraries for all F1 lines were constructed as described in a previous study [40]. Briefly, 500 ng of DNA template from each individual was double-digested using the restriction enzymes EcoRI and NlaIII (20 U/ reaction; New England Biolabs, Ipswich, MA, USA) in a combined reaction for 30 min at 37 °C. Subsequently, each fragmented sample was purified using a Qiagen MinElute Reaction Cleanup Kit (Qiagen, Valencia, CA, USA) and eluted in 20 μL of elution buffer (EB). The fragments were then ligated to P1 (includes a unique 4- to 8-bp multiplex identifier used to distinguish each individual) and P2 adapters that bound to the EcoRI and NlaIII overhangs, respectively. In each 40-μL reaction, 500 ng of DNA, 1 μL of P1 adapter (10 mM), 1 μL of P2 adapter (10 mM), 1 μL of T4 ligase (1000 U/mL), 4 μL of 10X T4 ligation buffer, and double-distilled water were mixed. The ligation was performed in a PCR machine using the following conditions: 37 °C for 30 min, 65 °C for 10 min, followed by a decrease in temperature to 20 °C at a rate of 1.3 °C/min. The samples were pooled and size-selected (400–600 bp) from an agarose gel. The DNA product was subsequently purified using a Qiagen MinElute Gel Purification Kit and eluted in 10 μL of EB. The paired-end (150 bp) sequencing of the ddRAD products from the 202 individuals was performed using the Illumina HiSeqXten sequencing platform (Illumina, Inc., San Diego, CA, USA). The sequencing data for each individual was extracted according to the specific multiplex identifier.

### SNP discovery and genotyping

We first filtered out Illumina short reads lacking sample-specific multiplex identifiers and the expected restriction enzyme motifs. Thereafter, the reads were filtered on the basis of their quality scores using Trimmomatic (v0.32) [58] in three steps: (1) removal of adapters; (2) removal of reads with bases from the start or end of a read, if below the quality threshold of 3; and (3) scanning of the reads with a 4-bp sliding window, removing reads with an average Phred quality per base below 15.

The STACKS pipeline (Version 1.32) [59] was used to assemble the loci, de novo, from the sequencing data for SNP calling. The USTACKS, CSTACKS, SSTACKS, and GENOTYPE programmes were then used to create libraries of the loci, i.e., one for each individual and one for all the loci shared among the individuals. The minimum depth of coverage required to create a stack was 3; the maximum distance (in nucleotides) allowed between stacks was 3; 2 mismatches were allowed between sample loci when the catalogue was built. The detailed parameters used were as follows: USTACKS: -t gzfastq -i -m 3 -M 3 -p 15 -d -r –f –o; CSTACKS: -b 1 –o –s –n 2 –p 15; SSTACKS: -b 1 –c –p 15; GENOTYPE: -b 1 –P -r 1 -c -s -t CP.

Only the SNPs with miss rates (number of samples with no genotype information/number of total samples) less than 10% along with biallelic SNPs were selected using custom PERL scripts (https://github.com/Niuyongchao/Fish_linkage_map) to avoid sequencing errors.

### Linkage map construction

A linkage map was constructed using JoinMap 4.1 [60]. The linkage group assignments were made under a logarithm of odds (LOD) score limit of 5.0–7.0. The regression mapping algorithm and Kosambi’s mapping function were used for map construction with the following settings: Rec = 0.4, LOD = 1.0, Jump = 5. The resulting linkage maps were drawn using a custom PERL script (https://github.com/Niuyongchao/Fish_linkage_map).

### Comparative genome analysis

Using a method from a previous study [61], comparative mapping was performed between our genetic map and the reference genomes of three Siluriformes fish, including the channel catfish (*I. punctatus*) and the yellow catfish (*P. fulvidraco*). In brief, the ddRAD tag markers were mapped to the two catfish genomes using the BLAST software suite (NCBI BLAST+ version 2.6.0) with an e-value cut-off of 10^− 10^. In cases where the search of a query sequence hit two or more loci, the hit with the smallest e-value was considered significant [38]. Finally, markers with a single unique position in the three reference genomes were used to perform comparative mapping and the genomic synteny was visualized using Circos software.

### QTL mapping

The QTLs were identified using MapQTL 6.0 with an interval mapping algorithm [62]. Automatic cofactor selection (backward elimination, *P* < 0.05) was used for the detection of significantly associated markers as cofactors. The LOD significance threshold levels were determined by a permutation test on the basis of 1000 permutations at a significance level of *P* < 0.05. The location of each QTL was determined according to its LOD peak location and surrounding region. The percentage of the phenotypic variance explained by a QTL (R^2^) was estimated at the highest probability peak. The QTL results were drawn using a custom PERL script (Genepioneer Biotechnologies, Nanjing, China). QTLs with LOD scores exceeding the chromosome-wide (CW) LOD threshold at *P* < 0.05 were considered as suggestive, while those with LOD scores exceeding the genome-wide (GW) LOD threshold at *P* < 0.05 were considered as significant.

### Kompetitive allele specific PCR assay

To confirm and refine the positions of the significant GW QTLs detected in the F1 mapping population, the 200 remaining individuals from the 400 total offspring of a full-sib family at 105 days post-hatch were used. In addition, in order to avoid the restriction of the remaining population on the genotyping of the parental fish, the sex marker associations were tested in the two parents of the 400 offspring (1 ♀:1 ♂) and in 48 independent wild individuals (24 ♀:24 ♂) originating from the Yangtze River, Yellow River, Amur River and Pearl River. Marker phenotype association was tested using KASP assays based on SNPs that were commonly found in the mapping families and spanned the regions of 8 significant GW associations with weight (un_15322259, un_40276574, un_36488182, un_47032303, un_34909841, un_54585281), body proportion (un_16957051), sex determination (un_42441442, un_40145170, un_43077153, un_47032303, un_36488182, un_15322259, un_40276574, un_34909841, un_13604800, un_25544718, un_63006763, un_55377625), and hypoxia tolerance (un_28380227).

The KASP assay was carried out according to the manufacturer’s recommendations (LGC Genomics, Beverly, MA, USA) and a previous study [63]. Marker sequences and KASP assay primers are listed in Table S8. Amplification was carried out starting with 15 min at 94 °C, followed by 10 touchdown cycles of 20 s at 94 °C and 60 s at 65–57 °C and 26–35 cycles of 94 °C for 20 s and 60 °C for 1 min. Endpoint genotyping was done using the CFX Manager 3.1 software. The specificity and sensitivity of all tested markers are listed in Table S2. Afterwards, an association analysis was performed using the Kruskal-Wallis test with SPSS 22.0. The Kruskal-Wallis test statistic (K*) and asymptotic significance (*P* < 0.05) were applied in order to test the magnitude of associations between the SNP genotypes and phenotype.

### Identification of potential candidate genes

For those markers that were located in the confidence intervals of GW QTLs and mapped at a single position on the assembled genome of *P. fulvidraco*, their sequences were extended by adding 500 nucleotide sequences from each side of the genome. The extended markers were used to identify conserved regions in the genome of *P. fulvidraco* and the potential candidate genes were identified in the conserved regions based on the annotation information.

We identified a number of candidate genes including Semaphorin-7A (Sema7a), Signal-induced proliferation-associated 1-like protein 1 (Sipa1) and Corticosteroid 11-beta-dehydrogenase isozyme 2 (HSD11B2), which were possibly responsible for the rapid growth and hypoxia tolerance traits (see below). The relative expression levels of Sipa1 and HSD11B2 were detected between fast-, medium- and slow-growing *P. vachelli* (fast: 13.2 ± 1.7 g; medium: 9.2 ± 1.1 g; slow: 4.7 ± 0.6 g) by qRT-PCR. Each group had nine individuals and three fish were pooled to create a sample. The materials for the hypoxia experiment were the same as our previously published articles [64]. In brief, control fish were removed from three aquaria for direct organ dissection. Afterwards, the water was deoxygenated for 25–30 min by bubbling pure nitrogen gas in order to decrease the oxygen concentration from 6.8 mg/L to 0.7 mg/L. After the fish were subjected to hypoxia for 1.5, 4 and 6.5 h, fish were quickly removed for organ dissection. After hypoxia for 6.5 h, the bubbling of nitrogen gas was replaced by bubbling with air, and the oxygen concentration was returned to a normal level (6.8 mg/L) in the three aquaria after 25–30 min. During the reoxygenation for 1.5, 4 and 6.5 h, fish were quickly removed for organ dissection.

The harvested organs were immediately frozen in liquid nitrogen and stored at − 80 °C. To evaluate the role of Sema7a in response to hypoxia and reoxygenation in *P. vachelli*, the sampling of liver and brain tissues from nine fish of the control (C) and challenged groups (hypoxia for 1.5, 4 and 6.5 h: H1.5, H4 and H6.5, respectively; reoxygenation for 1.5, 4 and 6.5 h: R1.5, R4 and R6.5, respectively) in the three aquaria was also conducted. Three fish were pooled to create a sample from an aquarium. qRT-PCR with β-actin as an internal control was used to explore mRNA expression levels. qRT-PCR was performed with the SYBR Green Master kit according to the manufacturer’s instructions (Roche, Basel, Switzerland). The primers for qRT-PCR are listed in Table S8. The experiments were carried out in triplicate in an ABI Stepone™ Plus (Applied Biosystems, USA) with a total volume of 20 μL containing 10 μL of SYBR green mastermix, 4 μL of cDNA (500 ng), and 3 μL each of forward and reverse primers (2 μmol/L). The qRT-PCR was carried out as follows: 95 °C for 10 min, followed by 40 cycles of 95 °C for 15 s, and 55 °C for 1 min. The expression levels were calculated by the 2^-△△CT^ method and subjected to statistical analysis. During the analysis of mRNA expression, fold change was determined by comparing to the expression of a reference gene.

## Supplementary information

**Additional file 1: Table S1.** Phenotypic data, including growth-related trait values, phenotypic sex, and time to balance loss, for ddRAD-seq and SNP phenotypic associations.

**Additional file 2: Table S2.** Data production of ddRAD sequencing for each individual.

**Additional file 3: Table S3.** Polymorphism SNP markers and their associated sequence information.

**Additional file 4: Table S4.** Tabular representation of the integrated map of *Pelteobagrus vachelli*.

**Additional file 5: Table S5.** Genomic synteny visualized between LGs of *P. vachelli* with chromosomes of *P. fulvidraco* and *I. punctatus*.

**Additional file 6: Table S6.** Details of the KASP assay results.

**Additional file 7: Table S7.** All potential candidate genes for the 8 significant genome-wide QTLs.

**Additional file 8: Table S8.** List of the allele specific primers and common primers designed for the allele specific PCR genotyping assays, as well as basic information on the 12 markers.

## Data Availability

All data generated or analysed during this study are included in this published article and its supplementary information files. The raw sequence data from this study were deposited at the NCBI Sequence Read Archive (SRA) with the accession Number PRJNA542235.

## Abbreviations

QTL: Quantitative trait locus
MAS: Molecular marker-assisted selection
GSA: Genome scaffolding and assembly
CGA: Comparative genomic analysis
SNP: Single-nucleotide polymorphism
ddRAD-seq: Double digest restriction-site associated DNA sequencing
GW: Genome-wide
CW: Chromosome-wide
KASP: Kompetitive Allele Specific PCR
W: Weight
BL: Body length
CF: Condition factor
HL: Head length
TL: Total length
BW: Body width
BH: Body height
BW/ED: Eye distance
BH/ED: Eye diameter
SL: Snout length
CL: Caudal peduncle length
CH: Caudal peduncle height
